# Supplementary material for: Fusion of Dendritic Cells Activating Rv2299c Protein Enhances the Protective Immunity of Ag85B-ESAT6 Vaccine Candidate against Tuberculosis
Source: Pathogens. 2020 Oct 22;9(11):865. doi: 10.3390/pathogens9110865 (PMC7690420; doi:10.3390/pathogens9110865)
Supplement: Supplementary file 1 [file pathogens-09-00865-s001.pdf]

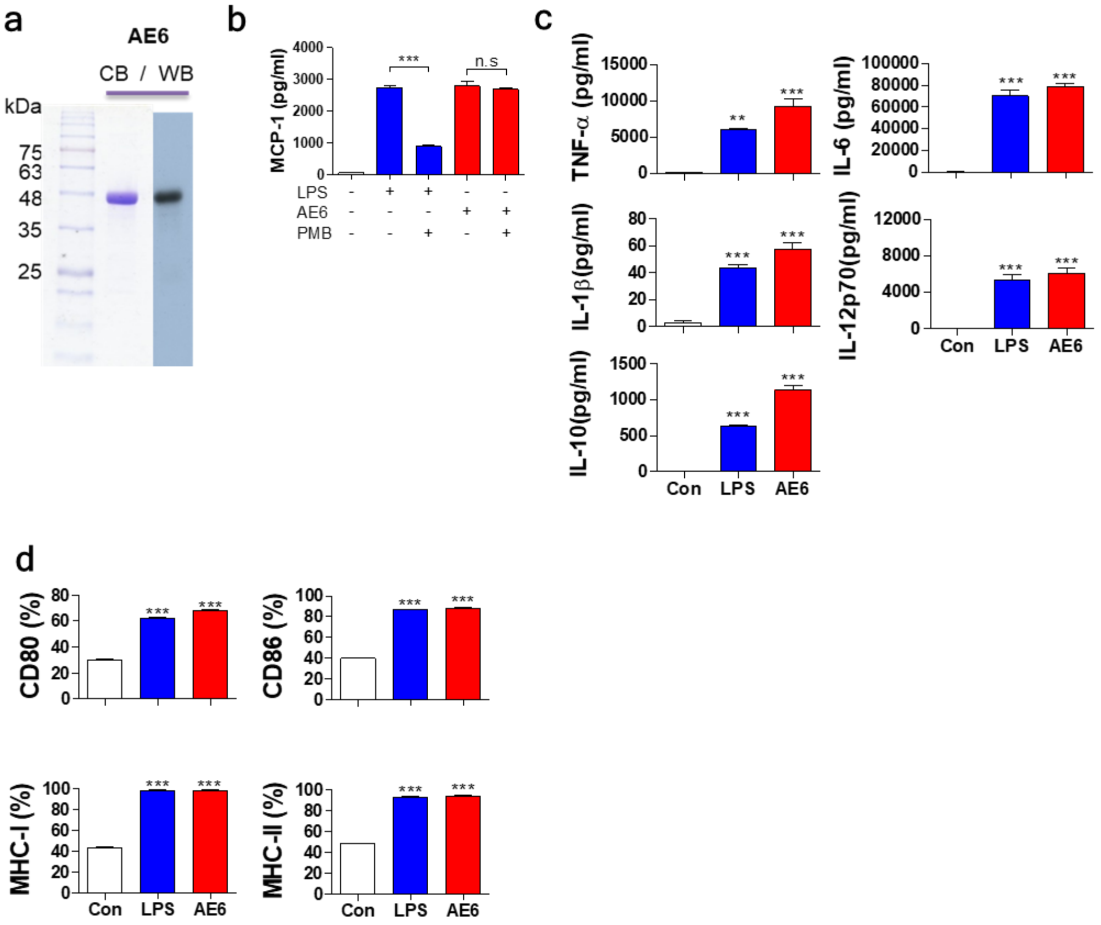

Figure S1. Recombinant Ag85B-ESAT6 induces maturation of dendritic cells (DCs) and Th1 responses. **(a)** Recombinant Ag85B-ESAT6 (AE6) was produced in BL21 cells and purified using Ni-NTA resin. The purified protein was subjected to sodium dodecyl sulfate-polyacrylamide gel electrophoresis (SDS-PAGE) and western blot analysis using a mouse anti-His antibody (Ab). **(b)** To ensure that Ag85B-ESAT6-induced RAW264.7 cells (5 × 104 cells) activation was not owing to endotoxin contamination in the protein preparation, Ag85B-ESAT6 (10 μg/mL) or lipopolysaccharide (LPS, 100 ng/mL) was pretreated with polymyxin B (20 μg/mL) for 1 h prior to stimulating the RAW264.7 cells cultures. After 24 h, the quantities of MCP-1 in the culture medium were measured by enzyme-linked immune sorbent assay (ELISA). All data are expressed as the mean values ± standard deviation (SD; $n = 3$); *** $p < 0.001$ = a significant difference compared to the Ag85B-ESAT6-treated RAW264.7 cells, as determined by unpaired Student's t-test. Treatments with no significant effect are indicated asn.s. **(c)** DCs were activated with the indicated concentration of Ag85B-ESAT6 or LPS (100 ng/mL) for 24 h. The TNF-, IL-1, IL-6, IL-10, and IL-12p70 levels in the culture medium were measured by ELISA. All data are expressed as mean ±SD ($n = 3$). The levels of significance (** $p < 0.01$, or *** $p < 0.001$ determined by one-way analysis of variance [ANOVA]) of the differences between the treatment data and the control data are indicated. **(d)** Activated DCs were stained with anti-CD80, anti-CD86, anti-MHC class I Ab, or anti-MHC class II Ab, and the expression of these surface markers was analyzed. The percentage of positive cells is shown in each panel. The bar graphs depict data as the mean ±SD ($n = 3$). The levels of significance (*** $p < 0.001$, determined by one-way ANOVA) of the differences between the treatment data and the control data are indicated.
